# Zinc Oxide Nanoparticles Induce Necrosis and Apoptosis in Macrophages in a p47phox- and Nrf2-Independent Manner

**DOI:** 10.1371/journal.pone.0065704

**Published:** 2013-06-03

**Authors:** Verena Wilhelmi, Ute Fischer, Heike Weighardt, Klaus Schulze-Osthoff, Carmen Nickel, Burkhard Stahlmecke, Thomas A. J. Kuhlbusch, Agnes M. Scherbart, Charlotte Esser, Roel P. F. Schins, Catrin Albrecht

**Affiliations:** 1 IUF - Leibniz Research Institute for Environmental Medicine, Düsseldorf, Germany; 2 Clinic of Pediatric Oncology, Haematology and Clinical Immunology, Centre of Child and Adolescent Health, Medical Faculty, Heinrich-Heine-University of Düsseldorf, Düsseldorf, Germany; 3 Interfaculty Institute for Biochemistry, University of Tübingen, Tübingen, Germany; 4 Air Quality and Sustainable Nanotechnology, Institute of Energy and Environmental Technology, Duisburg, Germany; Mayo Clinic, United States of America

## Abstract

In view of the steadily increasing use of zinc oxide nanoparticles in various industrial and consumer applications, toxicological investigations to evaluate their safety are highly justified. We have investigated mechanisms of ZnO nanoparticle-induced apoptosis and necrosis in macrophages in relation to their important role in the clearance of inhaled particulates and the regulation of immune responses during inflammation. In the murine macrophage RAW 264.7 cell line, ZnO treatment caused a rapid induction of nuclear condensation, DNA fragmentation, and the formation of hypodiploid DNA nuclei and apoptotic bodies. The involvement of the essential effector caspase-3 in ZnO-mediated apoptosis could be demonstrated by immunocytochemical detection of activated caspase-3 in RAW 264.7 cells. ZnO specifically triggered the intrinsic apoptotic pathway, because Jurkat T lymphocytes deficient in the key mediator caspase-9 were protected against ZnO-mediated toxicity whereas reconstituted cells were not. ZnO also caused DNA strand breakage and oxidative DNA damage in the RAW 264.7 cells as well as p47^phox^ NADPH oxidase-dependent superoxide generation in bone marrow-derived macrophages. However, ZnO-induced cell death was not affected in bone marrow-derived macrophages of mice deficient in p47^phox^ or the oxidant responsive transcription factor Nrf2. Taken together, our data demonstrate that ZnO nanoparticles trigger p47^phox^ NADPH oxidase-mediated ROS formation in macrophages, but that this is dispensable for caspase-9/3-mediated apoptosis. Execution of apoptotic cell death by ZnO nanoparticles appears to be NADPH oxidase and Nrf2-independent but rather triggered by alternative routes.

## Introduction

Nanotechnology is one of the key technologies of the current and upcoming decades, creating an enormous number of novel marketing potentials. Especially metallic nanoparticles offer great industrial opportunities due to their unique properties. Among these are zinc oxide nanoparticles (ZnO NP), which are produced in high tonnage and utilized in many commercial products. Because of their excellent UV-adsorbing properties and concurrent transparency for visible light, ZnO NP have found their use as efficient UV-protectors in cosmetics like sunscreens as well as in paints or finishing materials of building storefronts [1], [2]. Antibacterial properties of this material are used in household products like toothpaste or in food-packaging materials [3], [4]. In the fields of biotechnology and nanomedicine ZnO-based biosensors and biomedical nanomaterials containing ZnO are being developed for cancer treatment applications and improved drug delivery [5], [6]. The broad applicability of ZnO nanoparticles implies human exposure via different body entrance routes, including inhalation and ingestion. Macrophages are strategically located throughout the body tissues and play a central role in the defense against foreign material, dead cells and debris; these processes are implicated in both protective and adverse functions of macrophages in the regulation of the immune response in various pathogenic processes including inflammation and fibrosis [7]. Regarding particulate matter, macrophages are the most important cell type for uptake and clearance processes [8], [9], [10]. There is evidence that mononuclear cells, presumably the resident alveolar macrophages, mediate metal-related parenchymal disorders in occupational settings, such as metal fume fever which may result from inhalation of ZnO particles [11]. Investigations with crystalline silica dust have revealed a clear association between particle-induced apoptotic processes and the development of lung fibrosis [12].

Several recent studies have shown considerable cytotoxicity of ZnO NP to specific cell types, microorganisms and *in vivo* models [10], [13], [14], [15], [16], [17]. However, there are still a lot of controversies regarding the underlying pathways implicated in ZnO-induced cell death. This includes the impact of specific physicochemical properties of this material, like particle size and dissolution as well as the formation of reactive oxygen species (ROS) and the associated oxidative stress involving induction of lipid peroxidation and oxidative DNA damage [13], [14], [15], [18], [19], [20]. In professional phagocytes, such as macrophages and neutrophils, the dominant source of ROS is the classic nicotinamide adenine dinucleotide phosphate (NADPH) oxidase enzyme complex NOX2. Activation of this complex involves the recruitment and assembly of multiple cytosolic subunits including p47^phox^, p67^phox^ and p40^phox^ with its membrane-bound subcomplex consisting of gp91^phox^, p22^phox^ and Rac and results in the rapid generation of large amounts of superoxide anion (O_2_^−^) [21]. The NOX2-mediated oxidative burst represents a hallmark of the innate host defense to invading microorganisms. However, it is also strongly implicated in the adverse pulmonary effects of well-known particulate toxicants including asbestos and respirable crystalline silica dust [22], [23], [24].

Cells typically accomplish oxidative stress with the induction of antioxidant and detoxification enzymes mediated by the transcription factor nuclear factor erythroid-derived 2 related factor 2 (Nrf2) and hence may counteract the effects of necrosis and apoptosis-inducing triggers [25], [26], [27]. During oxidative stress, Nrf2 is released from its cytosolic repressor Keap1 and subsequently translocates into the nucleus where it binds to antioxidant response elements (ARE) residing within the promoter regions of many antioxidant and phase II genes [28]. The establishment of Nrf2 knockout mouse models has provided major support for the protective role of this pathway in ROS mediated cell death and diseases [29], [30]

The formation of ROS and associated induction of oxidative stress have been strongly linked to apoptosis in macrophages [31]. Apoptotic pathways induced by ROS-mediated DNA damage are fundamentally governed by mitochondria due to the release of apoptogenic factors out of the intermembrane space. The release of cytochrome c initiates the formation of the apoptosome, composed of Apaf-1 and the initiator caspase-9, resulting in caspase-9 activation. This complex in turn activates the executioner caspase-3, -6 and -7. Cleavage of their specific substrates leads to the typical apoptotic cellular changes and final cell death [32].

The aim of our study was to explore the mechanisms of ZnO-induced cell death in macrophages, as these cells are of key importance in uptake and clearance of particulates and orchestration of inflammation. Therefore, we investigated the necrotic and apoptotic effects of ZnO in the murine macrophage cell line RAW 264.7 as well as in primary macrophages obtained from bone marrow of p47^phox^ NADPH oxidase and Nrf2-deficient mice.

## Results

### ZnO induces cell death and apoptotic DNA damage with marginal impact of their specific surface area or solubilization

**Figure 1:**
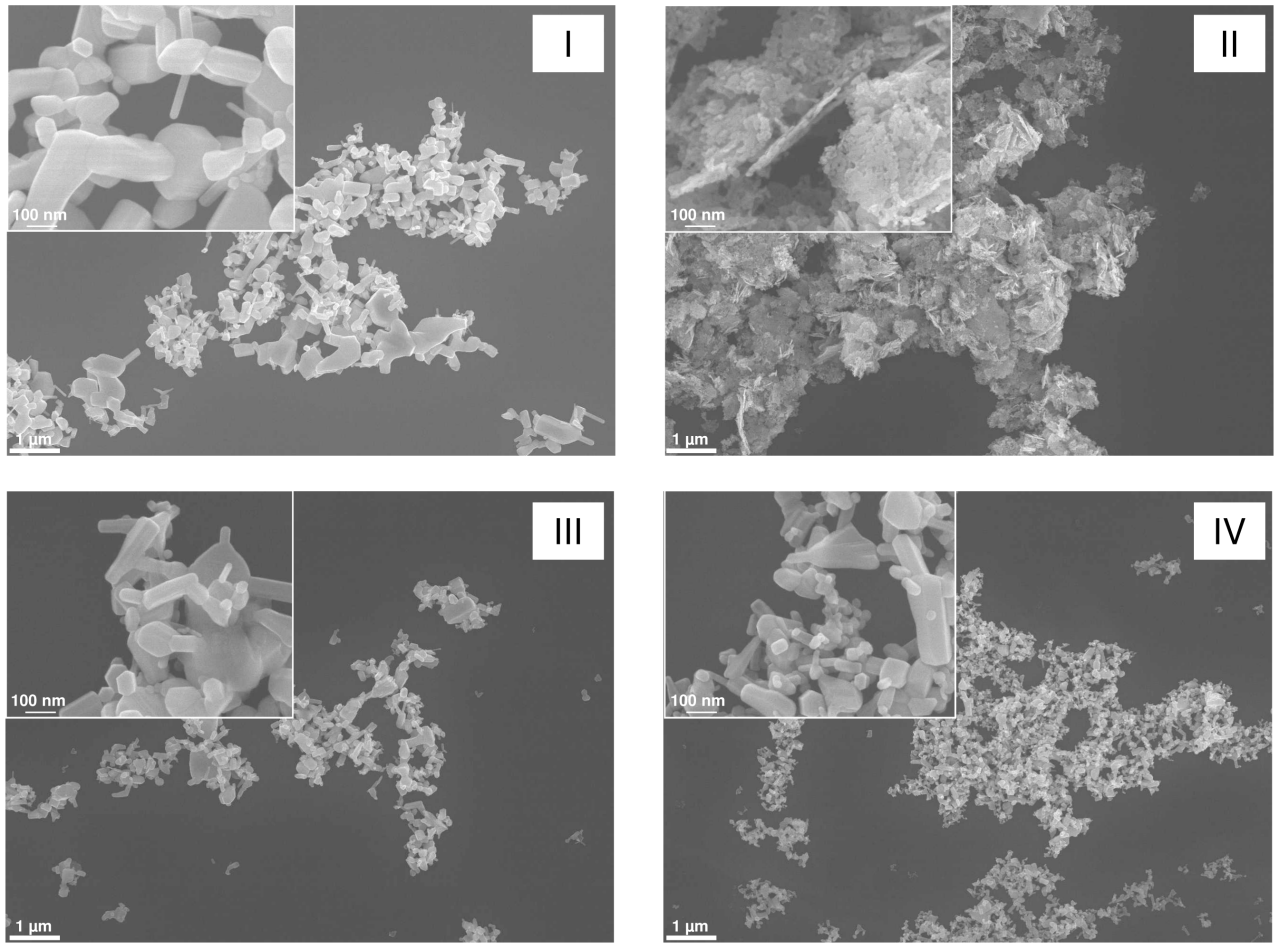
Representative Scanning Electron Microscopy images of the four ZnO samples used in present study. Samples were analyzed by a LEO (Zeiss) 1530 Scanning Electron Microscope at a magnification of 10,000× as well as 50,000× (inserts).

**Figure 2:**
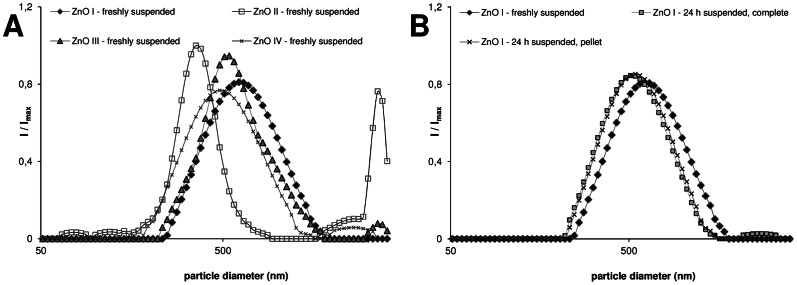
DLS curves of ZnO suspensions in complete cell culture medium. (A) Representative data of freshly prepared suspensions of the four ZnO samples. (B) Comparison of freshly suspended ZnO I versus complete suspension or resuspended pellet of ZnO I after 24 h incubation at 37°C. Data were obtained with a Beckmann Coulter Delsa Nano C.

**Figure 3:**
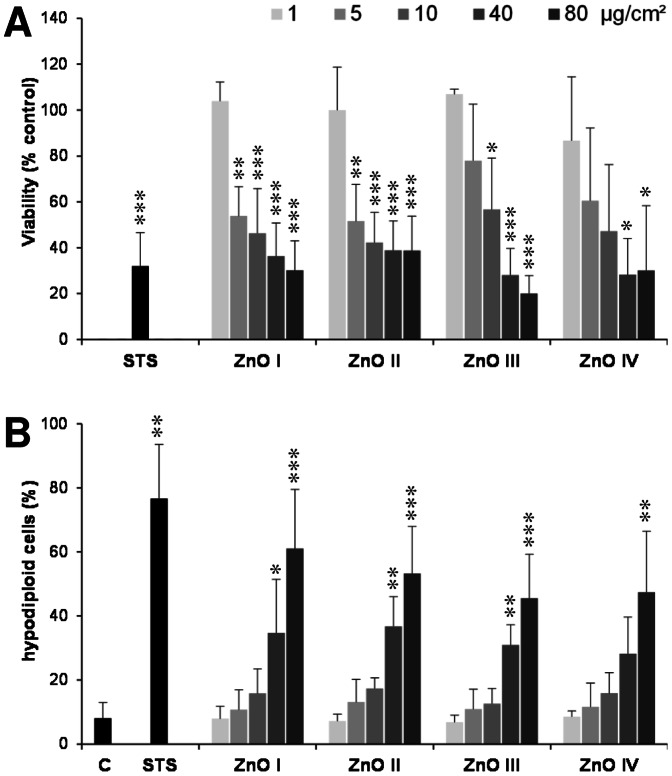
Cytotoxic effects of a panel of ZnO particles in RAW 264.7 macrophages. (A) Cell viability determined by WST-1 assay after 4 h treatment with four different ZnO particles at the indicated concentrations. Data are expressed as a percentage of the non-treated control cells, and represent three independent experiments (i.e. n = 3). As positive control, cells were treated for 4 h with 1 µM staurosporine (STS). (B) FACS analysis after 7-AAD staining revealing cells with hypodiploid DNA content after 4 h treatment with the panel of ZnO particles or staurosporine. Data are expressed as percentage of total cell events (n = 3).

Four representative ZnO nanoparticle samples (ZnO I–IV) were used to evaluate their cytotoxic effects as well as their specific apoptosis-inducing capacities in the RAW 264.7 macrophages. Despite their contrasts in primary particle size, morphological appearance (Fig. 1) and specific surface area (SSA) values, dynamic light scattering (DLS) analysis of the suspension of the four samples revealed a rather similar agglomeration/aggregation behavior (Table 1 and Fig. 2A). This was also reflected by a remarkably comparable mass dose-dependent decreases in cell viability by the samples as measured by the WST-1 assay (Fig. 3A). Analysis of the formation of hypodiploid cells, as a specific marker of apoptosis, revealed a dose-dependent increase reaching significance at the highest concentrations with all four ZnO samples (Fig. 3B). As all samples showed comparable results; therefore the sample ZnO I, labeled as ZnO in the following, was used exclusively for further investigations.

**Table 1:** Z.average, minimum and maximum values as well as polydispersity index (PDI) of different ZnO suspensions.

	Z. average (nm)	PDI	Min (nm)	Max (nm)
Complete cell culture medium	17	0.34	15	18
ZnO I, baked, freshly suspended	675	0.25	621	731
ZnO II, baked, freshly suspended	677	0.29	593	804
ZnO III, baked, freshly suspended	631	0.25	504	727
ZnO IV, baked, freshly suspended	593	0.24	488	695
ZnO I, baked, 24 h suspended, complete	617	0.25	585	656
ZnO I, baked, 24 h suspended, pellet	616	0.24	585	635
ZnO I, baked, 24 h suspended, supernatant	18	0.33	17	18

The suspension used for these analyses, were prepared in an identical manner to those that were used in the cell treatments (i.e. in complete cell culture medium and sonicated). Data represent three repeated measurements of two independent samples, and were obtained using a Beckmann Coulter Delsa Nano C.

To address the potential influence of solubilization of ZnO and the contributions of ionic Zn to apoptosis induction in the RAW 264.7 cells, effects of freshly prepared ZnO suspensions were compared to those of 24 h pre-incubated suspensions. Furthermore, we compared the effects of the respective particulate-free supernatants and pellets of those pre-incubated samples. The latter allowed for the evaluation of the relative contributions of particulate versus non-particulate, i.e. solubilized fractions. These respective preparations were also evaluated by DLS. As shown in Table 1 and in Fig. 2B, the measurements revealed that the agglomeration/aggregation status of the ZnO samples was not affected due to the 24 h pre-incubation. In addition, the measurements demonstrated that the prepared supernatants were indeed devoid of (nano)particulate ZnO.

**Figure 4:**
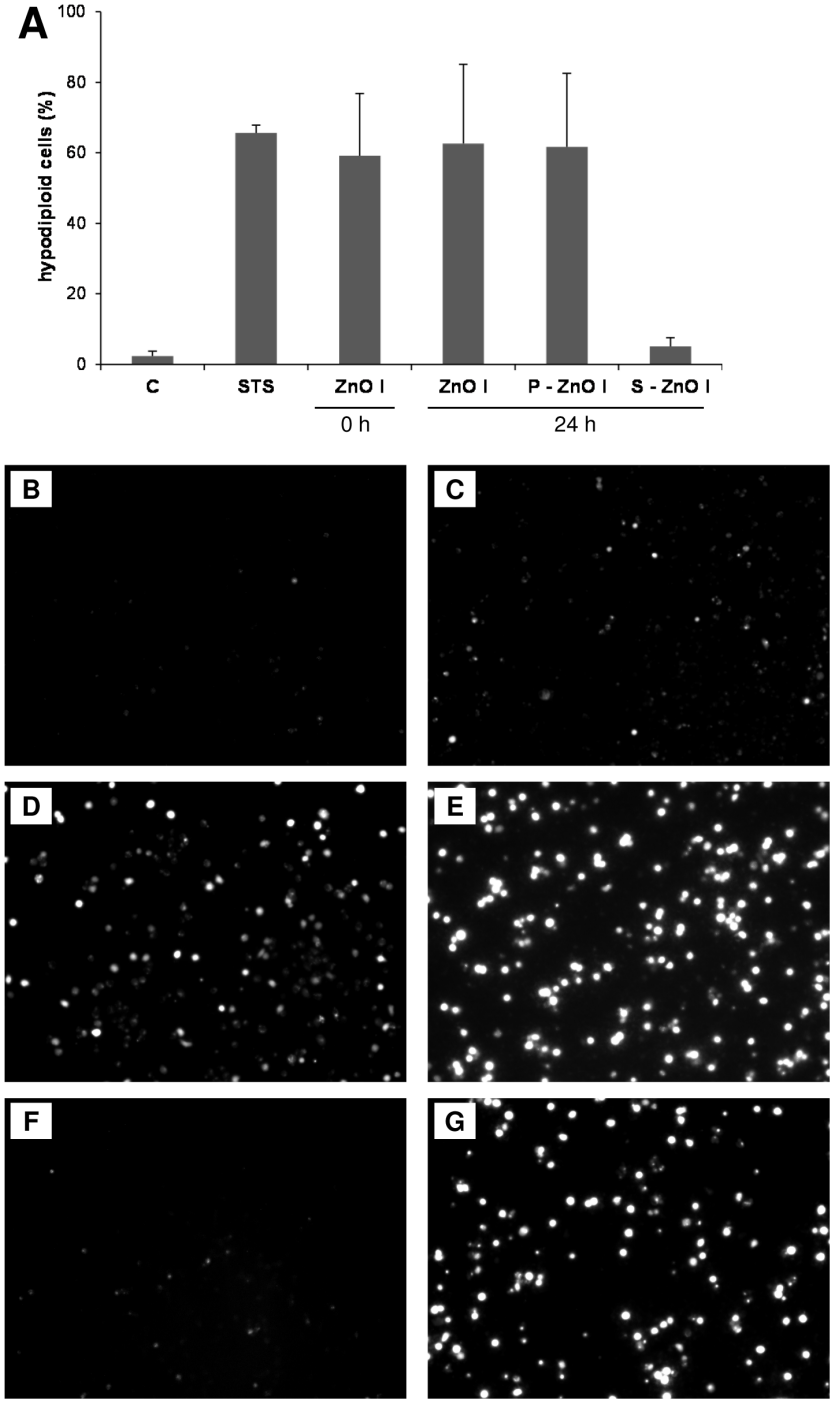
Investigation of the role of ZnO solubilization on the effects in RAW 264.7 cells. (A) FACS analysis of RAW 264.7 following a 24 h treatment with freshly prepared ZnO nanoparticles (ZnO I, 0 h), with ZnO suspension that were pre-incubated for 24 h at 37°C (ZnO I, 24 h), resuspended pellets of the 24 h pre-incubated suspension (P-ZnO I, 24 h) and the particle free supernatants collected after centrifugation of the pre-incubated suspension at 16.000 g (S-ZnO I, 24 h). All respective suspensions were prepared at 0.7 g/L, equaling the final treatment dose of 80 µg/cm^2^. Staurosporine (STS, 24 h, 0.1 µM) was used as positive control. Data are expressed as percentage of total cell events (n = 2). Detection of Zn using a fluorescent indicator in RAW 264.7 cells treated with control medium (B), treated for 0 h with ZnO particles 80 µg/cm^2^, freshly suspended (C), treated for 4 h with 5 µg/cm^2^ (D) or 80 µg/cm^2^ (E) freshly suspended ZnO particles or treated for 4 h with supernatant (F) as well as pellet (G) of a 80 µg/cm^2^ ZnO suspension after preincubation for 24 h at 37°C. Original magnification 100×.

As indicated in Fig. 4A, the supernatant fraction did not trigger any considerable hypodiploid cell formation after 24 h treatment, whereas all other treatments were equally effective. The effects were remarkably comparable to that of the positive control staurosporine (24 h treatment), a classical inducer of apoptotic cell death.

For an independent verification of the role of solubilization in ZnO-induced apoptosis, the RAW 264.7 cell cultures were evaluated by fluorescence microscopy, with the use of FluoZinTM-3. Results are shown in Fig. 4B–G. A clear dose-dependent fluorescent staining could be observed after 4 h treatment with 5 and 80 µg/cm^2^ ZnO. The fluorescence of freshly prepared suspensions was comparable to that of the 24 h pre-incubated suspensions, in concordance with the hypodiploid cell formation. Similarly, a marked fluorescence could be detected in cells that were treated with resuspended pellets, but not with the supernatants of the pre-incubated ZnO suspension (see Fig. 4). In cultures that were stained immediately after ZnO treatment, fluorescence was also found to be minimal.

### ZnO nanoparticles trigger classical morphological signs of apoptotic cell death

**Figure 5:**
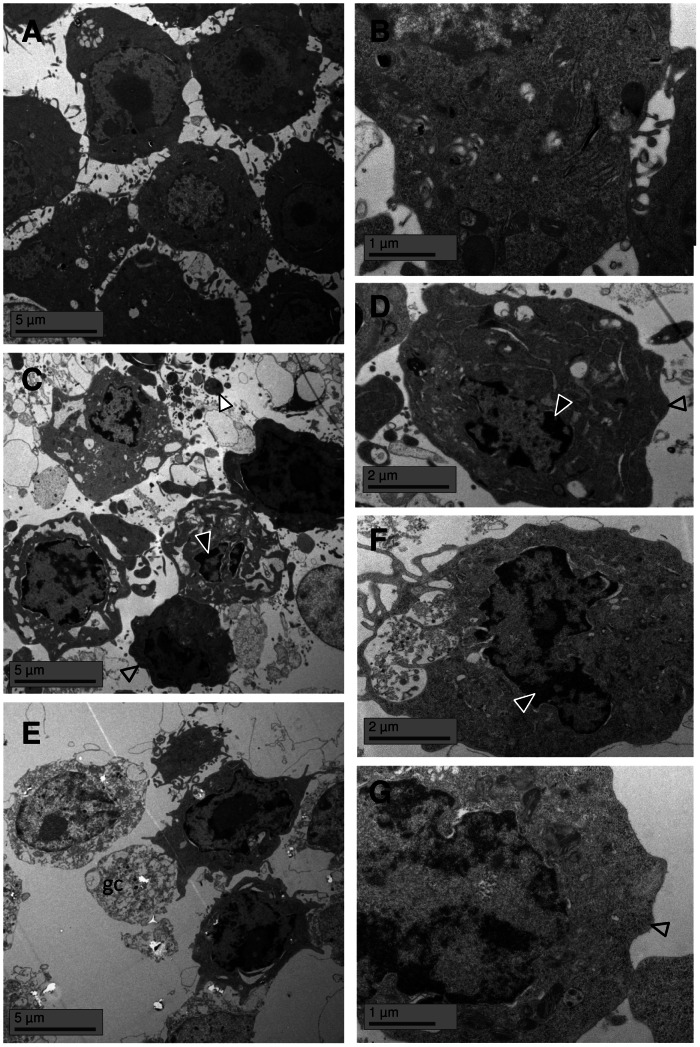
Transmission electron microscopy images of RAW 264.7 cells. Ultra-structural changes are shown after 40 µg/cm^2^ ZnO treatment for 4 h (E, F, G) compared to control cells (A, B) and staurosporine-treated cells (C, D). Nuclear condensation (black arrowhead), membrane blebbing (grey arrowhead), apoptotic bodies (white arrowhead), ghost cells (gc).

In recent years, there has been increasing concern about the potential introduction of artefacts in specific *in vitro* tests for the hazard assessment of nanoparticles [33], [34]. Accordingly, we also evaluated the ultrastructural changes in ZnO-treated RAW 264.7 macrophages by transmission electron microscopy to reveal characteristic morphological hallmarks of apoptotic cell death. In contrast to unaltered untreated control cells (Fig. 5A, B), staurosporine induced typical membrane blebbing, breakup of cells in apoptotic bodies and chromatin condensation (Fig. 5C, D). ZnO-treated RAW 264.7 macrophages revealed cells in different phases of cell death (Fig. 5E). In addition to brightly appearing “ghost” cells which were completely dismantled and without any clear shapes of cell compartments as clear signs of necrosis, less damaged cells showed embayed profiles and disaggregation due to apoptotic membrane blebbing. Higher magnification illustrates the deformation of the nucleus with condensed chromatin (Fig. 5F, G). Furthermore, phagocytotic processes in ZnO-treated cells were detected by the increased formation of pseudopodia which were closely connected to endocytotic vesicles.

### ZnO nanoparticles induce biochemical hallmarks of apoptosis in RAW 264.7 macrophages

**Figure 6:**
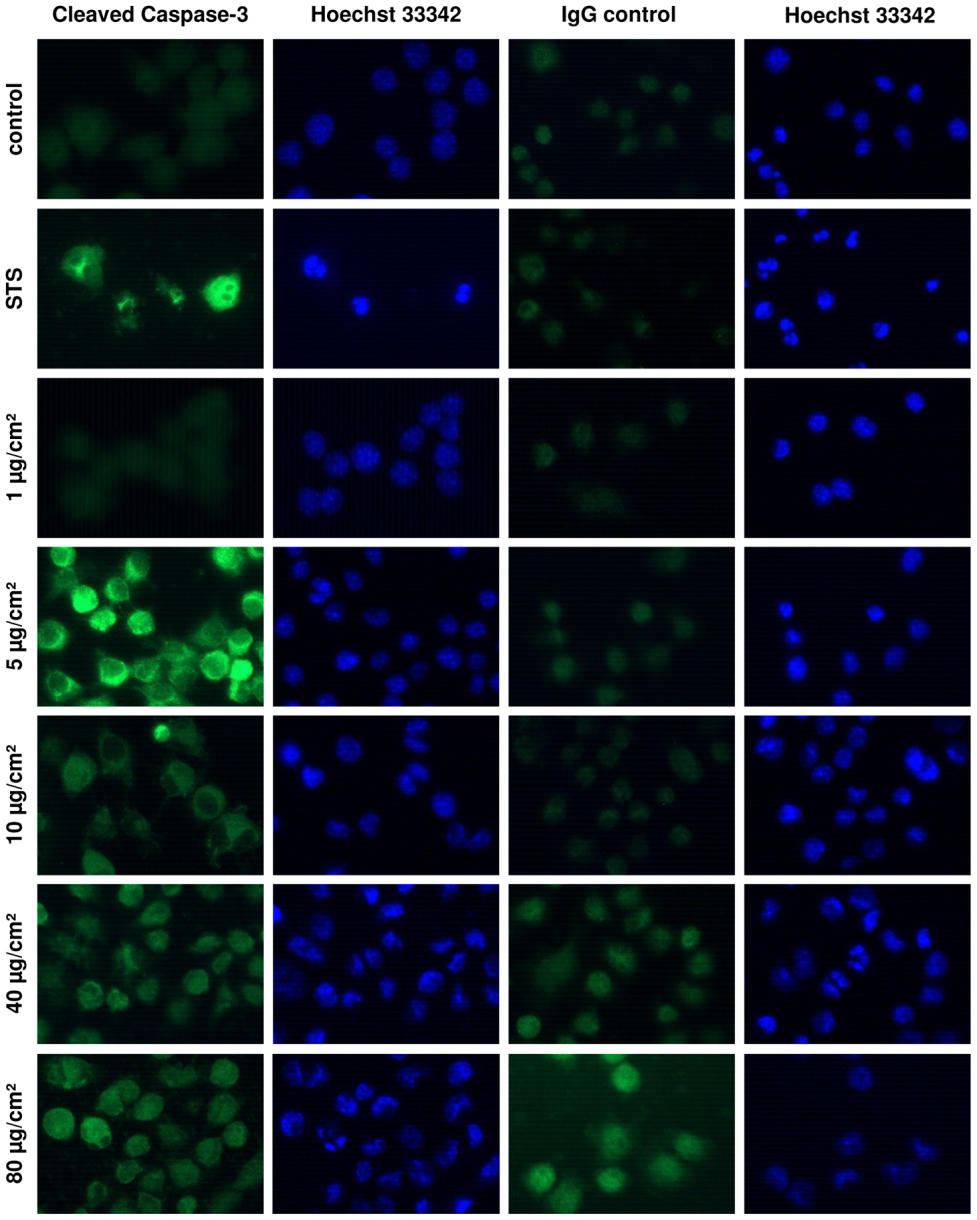
Activated caspase-3 and DNA staining in RAW 264.7 macrophages after 4 h treatment with ZnO or staurosporine (STS). Representative images are shown for activated caspase-3 (first column) and corresponding DNA staining with Hoechst 33342 (second column). To control for unspecific staining, CC-3 antibody was substituted by IgG antibody (third column) and shown with the corresponding Hoechst 33342 staining (fourth column). Original magnification 400×.

For the identification of classical apoptosis, the detection of activated executioner caspases is of key importance. Immunohistochemical labeling of cleaved caspase-3 revealed a clear signal in RAW 264.7 macrophages treated with 5 µg/cm^2^ ZnO, which in its intensity was comparable to the effect of the positive control staurosporine (Fig. 6). Concurrent DNA staining revealed nuclear chromatin condensation, which was seen neither in untreated cells nor in cells treated with lower ZnO concentrations. The staining procedure was also performed with unspecific IgG to allow for identification of potential false positive artefacts resulting from nonspecific antibody binding to ZnO particles and cellular structures or auto-fluorescence. This analysis revealed an increased dose-dependent non-specific signal for the two highest treatment concentrations (40 and 80 µg/cm^2^). Unlike caspase-3 activation which peaked at 5 µg/cm^2^ treatment, DNA fragmentation and nuclear condensation proceeded in a clear dose-dependent manner.

**Figure 7:**
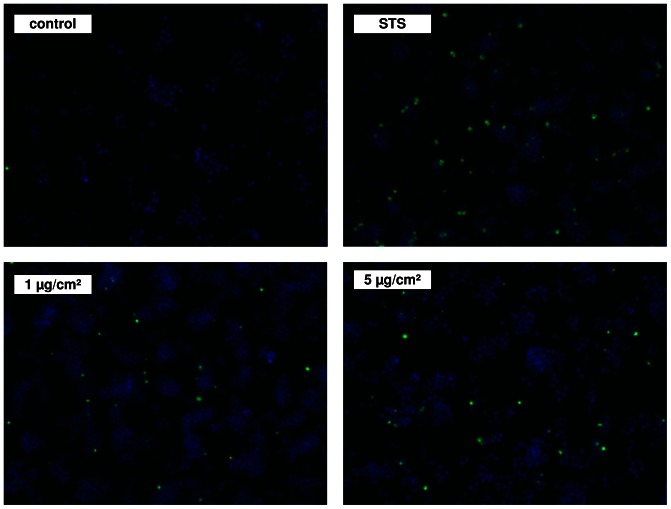
Fluorescent staining of RAW 264.7 macrophages after 4 h treatment with ZnO or staurosporine (STS) using TUNEL assay. Representative multichannel images are shown for apoptotic DNA fragmentation (green staining) and corresponding nuclei (blue staining). Original magnification 100×.

Apoptotic DNA fragmentation was evaluated by fluorescence microscopy using the TUNEL assay (Fig. 7). Treatment of RAW 264.7 macrophages with staurosporine or with 1 and 5 µg/cm^2^ ZnO revealed a significant appearance of positively labeled cells representing apoptotic DNA fragmentation.

### ZnO nanoparticles induce apoptosis via the intrinsic mitochondria-mediated pathway

**Figure 8:**
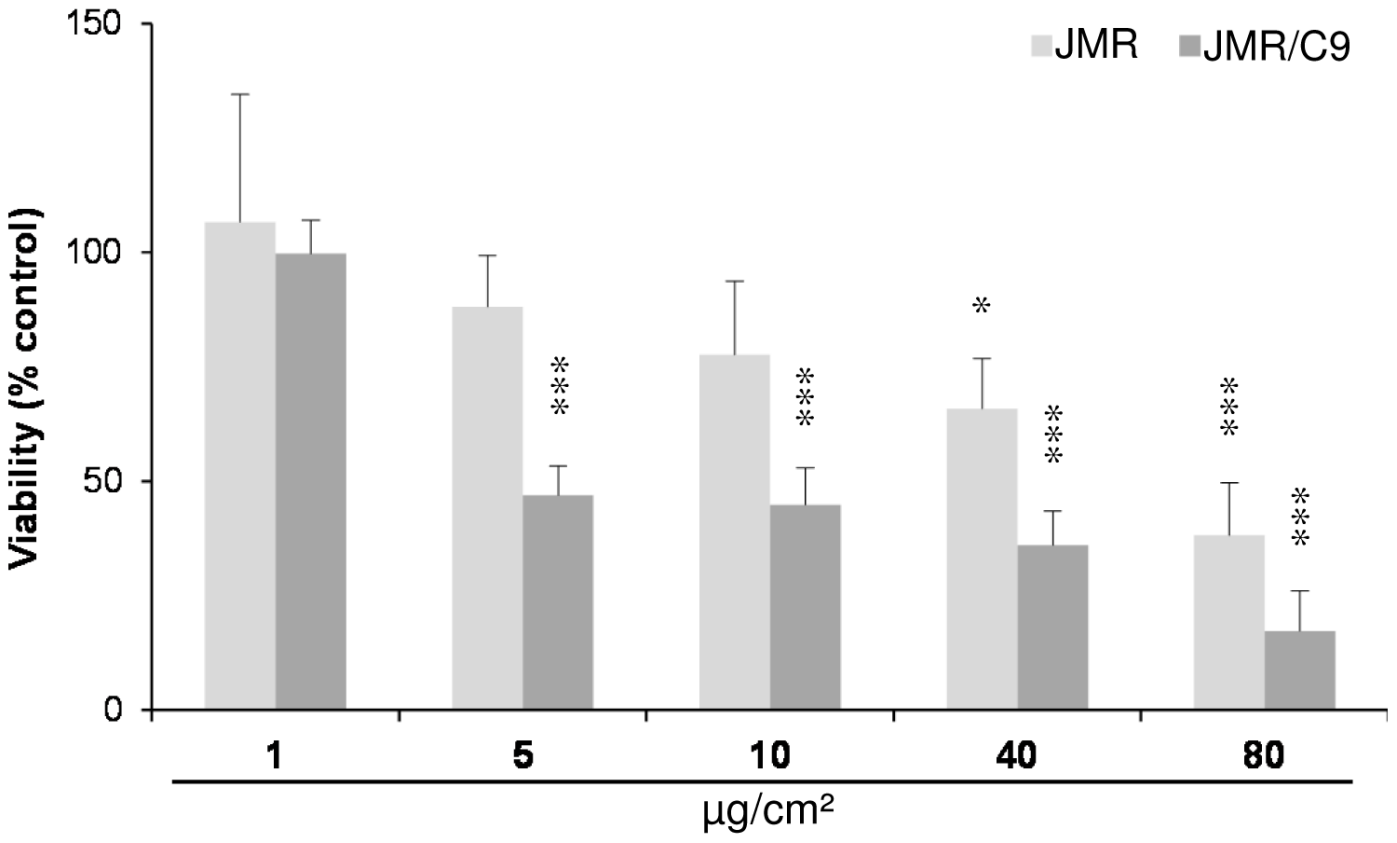
Cell viability (WST-1 assay) after 4 h of ZnO exposure observed in a mutant Jurkat T-cell line deficient in caspase-9 (JMR) versus their caspase-9-restored counterpart (JMR/C9). Data of treated cells are related to corresponding control cells (100% activity, n = 3).

For the investigation of the role of the intrinsic apoptotic pathway in ZnO-induced effects, a caspase-9-deficient Jurkat T-cell line (JMR), resistant to apoptosis, was used in comparison with caspase-9-restored JMR/C9 cells. Indeed, staurosporine was capable of causing a significant reduction of viability in the JMR/C9 cells, but not in the caspase-9 deficient cell line (Fig. 8). Following treatment with 5 or 10 µg/cm^2^ ZnO, the cell viability of JMR cells remained largely unaltered compared to the untreated control. In contrast, the viability of JMR/C9 cells dropped below 50% after the 4 h treatment with ZnO nanoparticles. At higher concentrations (40 to 80 µg/cm^2^) a dose-dependent cytotoxicity was observed in both cell lines.

### ZnO induces DNA strand breakage and oxidative DNA damage induction

**Figure 9:**
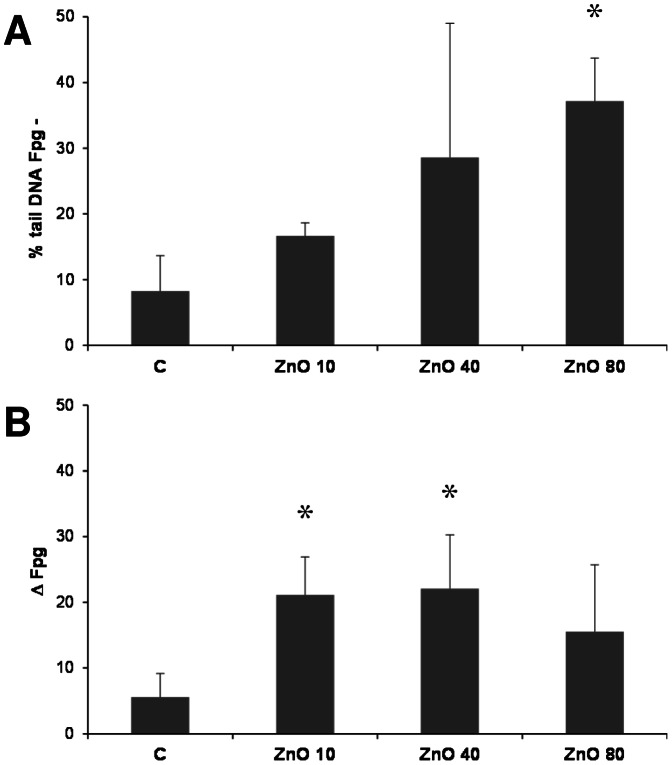
DNA damage in RAW 264.7 macrophages after 4 h treatment with ZnO as evaluated by the fpg-modified comet assay. (A) Percentage of DNA strand breakage measured as % tail intensity in the absence of fpg-enzyme (n = 3). (B) Oxidative DNA damage induction as determined the presence of fpg-enzyme (n = 3). Oxidative DNA damage is shown as the differences in % tail DNA as measured in the presence or absence of the Fpg enzyme: delta (Δ)Fpg = [% tail DNA+Fpg]−[% tail DNA-Fpg].

In order to investigate ZnO-induced oxidative stress and DNA damage, the formamidopyrimidine glycosylase (Fpg)-modified comet assay was used. DNA strand breakage by ZnO could be observed in a clear dose-dependent manner (Fig. 9A). Addition of Fpg, the murine homologue for the human DNA repair enzyme oxoguanine glycosylase, in the comet assay enables detection of oxidative DNA lesions, such as 8-hydroxydeoxyguanosine. Hence, this measurement can be considered as a sensitive marker of oxidative stress [35]. The Fpg inclusion also demonstrated a specific induction of oxidative DNA damage after ZnO treatment at 10, 40 and 80 µg/cm^2^ albeit in the absence of a clear dose dependency (Fig. 9B). Pre-treatment of the RAW 264.7 cells with the oxidative stress-inducing photosensitizer Ro 19-8022 followed by light exposure resulted in 15.8±1.0 (% tail DNA Fpg-) and 30.2±5.7 (Δ Fpg, data not shown).

### ZnO toxicity in mouse macrophages is NADPH oxidase and Nrf2-independent

**Figure 10:**
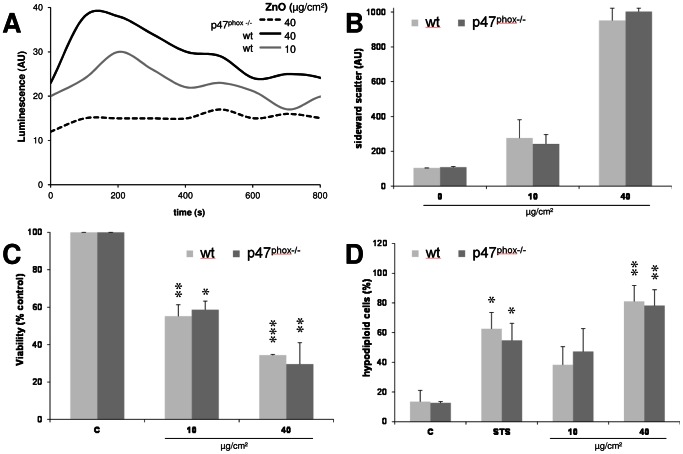
Effects of ZnO exposure in murine bone marrow-derived macrophages of p47^phox−/−^ versus wt animals. (A) Superoxide detection via lucigenin-amplified chemiluminescence depicted in arbitrary units (AU). (B) FACS analysis of sideward scatter related granularity to investigate particle uptake (n = 2). (C) Cell viability determined by WST-1 assay (n = 2). (D) Content of hypodiploid cells determined by FACS analysis after 7-AAD staining (n = 2). STS: staurosporine.

**Figure 11:**
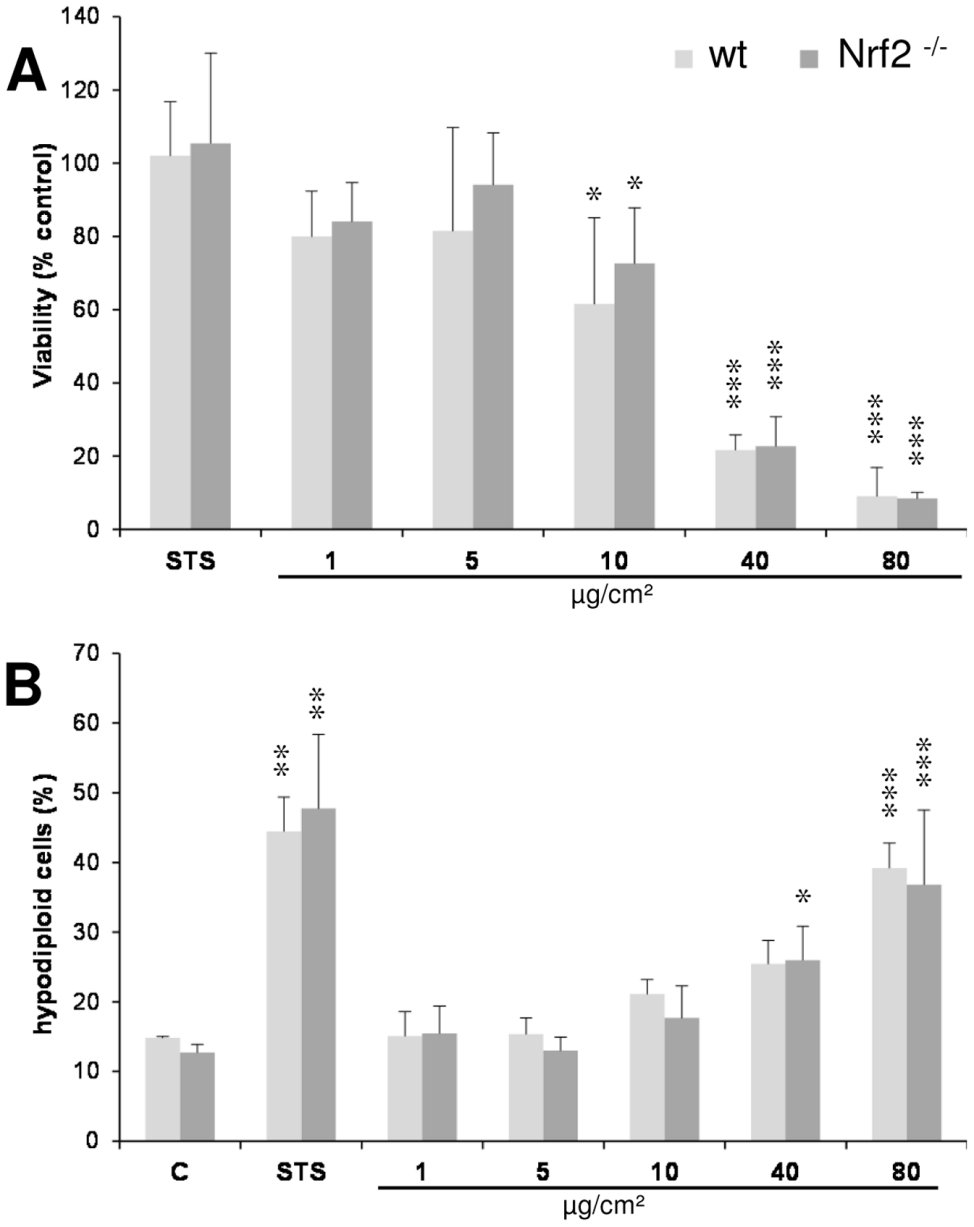
Bone marrow-derived macrophages of Nrf2^−/−^ versus wt animals were investigated following 4 h ZnO treatment. (A) Cell viability (WST-1 assay) (n = 3). (B) FACS analysis to detect the percentage of cells with hypodiploid DNA after 7-AAD staining.

To address the role of the phagocyte-specific ROS production in ZnO-triggered cell death, experiments were performed with bone marrow macrophages derived from p47^phox−/−^ mice along with the macrophages obtained from C57B6/J wild type (wt) mice. Using lucigenin-amplified chemiluminescence, enhanced ROS formation could be observed in ZnO-treated macrophages from wt mice, whereas this formation was impaired in the treated macrophages from the p47^phox−/−^ animals (Fig. 10A). The uptake of ZnO particles by the respective macrophages was evaluated by FACS analysis. Analysis of the sideward scatter (SSC) evidenced no differences in ZnO particle uptake between wt and p47^phox−/−^ macrophages (Fig. 10B). WST-1 assay and hypodiploid DNA content analyses revealed no differences in the effects of ZnO in macrophages from p47^phox−/−^ versus wt mice (Fig. 10C and D). In subsequent experiments we addressed the role of the redox-sensitive transcription factor Nrf2 using bone marrow-derived differentiated macrophages from Nrf2^−/−^ mice and wt littermates. Clear dose-dependent effects of ZnO were observed, however, neither in the WST-1 assay nor in the hypodiploid DNA content measurement assay differences were observed in relation to the genetic background of the macrophages (Fig. 11A and B).

## Discussion and Conclusions

In the present study, zinc oxide nanoparticles caused NADPH-dependent oxidative burst and apoptotic responses, but both were not in a causal relation. Neither NADPH-dependent superoxide formation nor a master regulator of the antioxidant response, Nrf2, were involved in ZnO-induced apoptosis in RAW 264.7 macrophages. Apoptosis was apparent already within 4 h of treatment, in concordance with previous investigations in our laboratory [33]. A selection of four representative ZnO samples of varying primary particle size and specific surface area (SSA) revealed a remarkably similar impact on cytotoxicity and DNA fragmentation. As such, our present findings suggest that the toxic properties of ZnO towards macrophages occur merely independent of these physicochemical properties. However, the behavior of the four different samples under cell culture conditions appeared to be rather similar. In all cases, considerable agglomeration was observed, which can be explained by the presence of abundant protein in the FCS-containing culture medium (e.g. [35], [36], [37]).

The apoptosis-inducing effect of ZnO in the RAW 264.7 cells was apparent by the occurrence of classical morphological and biochemical hallmarks of apoptotic death, such as nuclear condensation, apoptotic blebbing, and activation of caspase-3. Employing caspase-9-deficient and proficient Jurkat T cells, the involvement of the mitochondrial apoptotic pathway could be demonstrated. Our current observations are in line with recent studies showing activation of caspase-3 and -9 by ZnO nanorods in A549 human lung epithelial cells [20] and activation of caspase-3, DNA fragmentation and phosphatidylserine exposure by ZnO in dermal fibroblasts [38]. In these cell lines, ZnO induced DNA damage response was shown to involve increased activation of p53 and p38 mitogen-activated protein kinase, which mediate cell cycle arrest, DNA repair or apoptosis induction [20], [38]. In our study, ZnO induced apoptosis in RAW 264.7 cells could be observed already after 4 h treatment. Others have shown involvement of p53-signaling in the apoptosis for this cell line after 12 to 24 hours (e.g. [39]). Moreover, we observed Caspase 9-dependent cell death in Jurkat cells. In the p53 gene of this cell line, missense and nonsense mutations have been identified ([40]). In our current study great care was taken to avoid assay interferences that may have led to false outcomes. Previously, we identified ZnO-mediated quenching artefacts when employing a commercial chemiluminescence kit for the detection of caspase-3 activity [33]. In the present study, performing of an immunocytochemical staining of cleaved caspase-3 revealed to some extent particle-related artefacts in the form of non-specific staining. However, this problem only occurred at the highest treatment concentrations (i.e. 40 and 80 µg/cm^2^), while the strongest caspase-3 activity was observed at an artefact-free concentration of 5 µg/cm^2^. The induction of apoptotic effects by ZnO at these lower treatment concentrations was confirmed by microscopical evaluation of DNA fragmentation. Noteworthy, also in this assay an impaired DNA fragmentation detection was found at the highest treatment concentrations, likely as a consequence of a quenching artefact. Taken together, this again emphasizes the necessity to use multiple assays for apoptosis validation by nanoparticles, as demonstrated in our previous study [33].

The caspase-9 proficient Jurkat T lymphocytes and RAW 264.7 macrophages displayed a remarkably similar sensitivity towards the toxic effect of the ZnO nanoparticles in the WST-1 assay. This may reflect the requirement of the mitochondrial pathway for efficient apoptosis induction [41]. In this progression, mitochondrial outer membrane permeabilisation causes cytochrome c release followed by the assembly of the caspase-9 containing apoptosome and subsequent activation of the executioner caspase-3, -6 and -7. Cleavage of their specific substrates leads to the typical changes associated with apoptosis within the cell [32]. One of the first targets of active caspases are the permeabilised mitochondria themselves, leading to a decline in ATP levels, loss of mitochondrial transmembrane potential, and the production of ROS [42]. However, the presently observed loss of viability at the higher ZnO concentrations in caspase-9-deficient Jurkat cells suggests an involvement of other non-apoptotic pathways. In concordance with this, our electron microscopical findings of neighbored ZnO-treated macrophages indicate heterogeneity of cell-damaging effects. Observations ranged from mildly affected cells to cells with more clear signs of apoptotic processes like nuclear condensation and membrane blebbing, as well as to completely disassembled remains of necrotic “ghost cells”.

The observed variations in the cellular response may depend on the individual differences in cellular dose, on various routes of uptake mechanisms under participation of different receptors, on the respective cell cycle phase and/or the rate of ZnO dissolution. In the literature, there is a controversy regarding the impact of ZnO solubility on its toxic effects. Xia and colleagues [13] discussed dissolution of ZnO in culture media and subsequent formation of ROS as a major mechanism of toxicity in both RAW 264.7 cells and BEAS-2B bronchial epithelial cells. Indeed, in concordance with our electron microscopy analysis, Xia et al. also reported that ZnO could not be detected intracellularly, in contrast to CeO particles. However, we observed no difference in the induction of hypodiploid DNA by freshly prepared and pre-incubated ZnO suspensions as well as resuspended pellets of the pre-incubated suspensions while particle free supernatants were inactive in the macrophages. An alternative mechanism, also forwarded by the findings of Xia et al. with RAW 264.7 cells [13], is that ZnO triggered apoptosis involves its rapid dissolution within the acidic lysosomes of the macrophages after their uptake in their particulate, agglomerated form. In further support of this, our subsequent experiments with the fluorescent probe FluoZinTM-3, revealed a marked and similar intracellular staining upon treatment with ZnO suspensions or with resuspended pellets of pre-incubated ZnO suspensions, whereas their supernatants caused negligible staining.

In the present study we did not address the potential role of ionic Zn by means of macrophage treatments with zinc salts. Interestingly, in this regard, it was recently demonstrated that in culture media containing phosphate and carbon ions addition of soluble Zn^2+^ causes formation of potentially toxic nanoparticulate zinc consisting of Zn-phosphate-carbonate, and hence may not serve as an appropriate non-particulate control [43]. Our present findings do not prove that dissolution may not be involved in ZnO toxicity. However, a contribution of a fraction of rapidly dissolved ZnO can be excluded for our experimental conditions. The observed differences between our findings and those from Xia et al. might relate to the different physicochemical properties of the ZnO sample used in their experiments, which was synthesized by flame spray pyrolysis [13]. Our findings on the lacking role of non-particulate extracellular zinc are also in line with findings by various other investigators. Using a transwell membrane system, Moos and colleagues demonstrated that the toxicity of ZnO particles towards a colon cell line was independent of the amount of soluble Zn in the culture medium and required a direct particle-cell contact [44]. In A549 lung epithelial cells, ZnO-induced ROS production was also described as a particle-mediated effect [45]. Lastly, in a recent study with RAW 264.7 cells, particulate ZnO was shown to induce apoptosis and, at high concentrations also necrosis, while dissolved ions were found merely to induce metallothionein synthesis [46].

Generation of ROS and induction of oxidative stress with subsequent perturbation of mitochondria and apoptosis was previously shown to be triggered by ZnO nanoparticles in RAW 264.7 macrophages [13] as well as in other cell types [15], [44]. In concordance with these findings, we found a concentration-dependent oxidative DNA damage induction in ZnO-treated RAW 264.7 cells using the Fpg-modified comet assay. Such oxidative DNA damage induction is closely related to the action of ROS and hence generally considered as a sensitive marker of oxidative stress [35]. The oxidative DNA damage observed at higher doses of ZnO was nearly as strong as the effect that could be generated in the macrophages by the photosensitizer Ro 19-8022 (data not shown). Moreover, we could demonstrate that ZnO triggers a concentration-dependent formation of superoxide radicals in bone marrow-derived macrophages from C57Bl/6 wild type mice but not p47^phox^ knockout animals. This demonstrated that the phagocytic NADPH oxidase enzyme complex NOX2 is a major source of ROS in ZnO-exposed macrophages. Receptor-dependent phagocytosis of pathogens by macrophages is a well described mechanism of rapid NADPH oxidase activation and superoxide formation known was respiratory burst [21]. Therefore, we also included ZnO uptake measurements in wt versus p47^phox^-deficient macrophages. Neither an impairment of uptake of ZnO particles nor a reduction of hypodiploid DNA formation was found in the p47^phox−/−^ model. As such, our findings indicate that p47^phox^ NADPH oxidase-mediated ROS generation is not directly implicated in ZnO-triggered apoptosis in macrophages.

To further explore the role of oxidative stress and a possible involvement of NADPH oxidase-independent sources of ROS like mitochondria, ZnO effects were also evaluated in primary macrophages from mice proficient or deficient in the oxidant-inducible transcription factor Nrf2, a master regulator of antioxidant response [47]. In a recent study, Nrf2 has been implicated in the DNA-damaging and apoptotic effect of silver nanoparticles in an ovarian carcinoma cell line via a mechanism involving cytoprotection by the Nrf2-regulated antioxidant heme oxygenase-1 [27]. However, Nrf2 may also be implicated in ROS-independent mechanisms of apoptosis induction. In T cells, Nrf2 deficiency was shown to be associated with increased sensitivity to anti-Fas mediated apoptosis [48]. Moreover, an Nrf2 binding antioxidant responsive element was recently identified within the gene promoter of the antiapoptotic protein Bcl-2 [49]. However, in our study we could not detect any significant difference in sensitivity of the wild type versus the Nrf2- deficient macrophages towards ZnO-triggered cell death. Interestingly, also the effects of our positive control staurosporine did not differ between both genetic backgrounds. This indicates that ZnO and staurosporine may act in macrophages via similar oxidative stress-independent pathways. This may also explain for the apparent discrepancies as observed in an earlier study on gene expression patterns of ZnO NP exposed BEAS-2B lung epithelial cells where genes involved in oxidative stress were upregulated in contrast to those coding for antioxidant enzymes [18].

In conclusion, ZnO nanoparticles can trigger a rapid p47^phox^ NADPH oxidase-mediated superoxide radical generation, oxidative DNA damage induction, necrosis and caspase-9/3-dependent apoptosis in macrophages. However, the observed cell death effects appear to be independently of this major phagocytic oxidant generating pathway as well as of the oxidant responsive Nrf2 pathway. Further studies are needed to unravel the precise molecular mechanisms that drive ZnO-induced toxic responses.

## Materials and Methods

### Cells

The murine macrophage-like cell line RAW 264.7 (ATCC# TIB-71; American Type Culture Collection, Manassas, VA, USA) was cultured at 37°C in humidified 5% CO_2_ atmosphere in Dulbecco's modified Eagle's medium (DMEM, Sigma-Aldrich, Deisenhofen, Germany) supplemented with 4 mM L-glutamine, 1.5 g/l sodium bicarbonate, 4.5 g/l glucose, penicillin (100 U/ml)/streptomycin (0.1 mg/ml) and 10% fetal calf serum (Sigma-Aldrich).

Generation of the caspase-9 restored cell clone JMR/C9 from the mutant T-cell line JMR deficient in caspase-9 was originally described by Samaraj and colleagues [50]. Both cell lines were cultured in RPMI-1640 medium with 10% heat-inactivated fetal calf serum, 2 mM L-glutamine, 1.5 g/l sodium bicarbonate, 4.5 g/l glucose and penicillin/streptomycin. Culturing proceeded in a humidified 5% CO_2_ atmosphere at 37°C. Subculturing of all cell lines was performed to maintain cells growing in the logarithmic phase.

Bone marrow cells were obtained from p47^phox−/−^ and C57Bl/6J wild type (wt) mice (Taconic, Germantown, NY, USA), originally produced in the laboratory of S. M. Holland [51]. Bone marrow cells were also obtained from Nrf2^−/−^ mice and C57Bl/6J littermate controls (Riken BioResource, Tsukuba, Japan), originally produced in the laboratory of M. Yamamoto [52]. Animals were maintained according to the guidelines of the Society for Laboratory Animals Science (GV-SOLAS). After sacrificing, both hind legs of mice were freed of soft tissue attachments. The tips of femur as well as the tibia were cut off and flushed with sterile PBS supplemented with penicillin/streptomycin. Cell suspensions were centrifuged (200 g, 10 min, 4°C) and resuspended in culture medium RPMI-1640 (PAA, Cölbe, Germany) containing 10% FCS gold (PAA), 2 mM glutamine, penicillin/streptomycin and 0.1% mercaptoethanol. 10 ml of 5×10^6^ cells in suspension containing 10% of L929 cell supernatant were seeded in bacterial culture plates (10 cm, Greiner bio-one, Solingen, Germany). After three days of incubation at 37°C in a humidified 5% CO_2_ atmosphere, 10 ml of freshly prepared medium containing 10% M-CSF/GM-CSF containing L929 supernatant were adjusted. After six days, the adherent fraction of cells mainly contains macrophages and can be harvested by scraping the rinsed cell layer. Double staining of the macrophage markers CD11b-FITC (BD Biosciences, Heidelberg, Germany) and F4/80-PE (eBioscience, Frankfurt, Germany) identified 54–97% CD11b^+^/F4/80^+^ macrophages by FACS analysis using a FACSCalibur operating with CellQuestPro software (BD Biosciences).

### Particle characteristics and treatment of the cells

The general cytotoxicity studies in RAW 264.7 cells were performed with four different samples of ZnO particles, indicated as ZnO I (from Nanostructured and Amorphous Materials Inc., with a declared primary particle diameter of 20 nm and a specific surface area (SSA) of 50 m^2^/g), ZnO II (Nanoactive, from Nanoscale Materials Inc., with a declared crystallite size of <10 nm and SSA of 70 m^2^/g), ZnO III (from Sigma-Aldrich, with a declared SSA <10 m^2^/g), and ZnO IV (obtained through the European Commission Joint Research Centre (Ispra, Italy) as reference nanomaterial NM-110, with a diameter of 100 nm and SSA of 14 m^2^/g). Samples ZnO I and ZnO II were kindly provided by Dr. R. Duffin (Edinburgh), sample III was directly purchased from Sigma-Aldrich and sample IV was obtained through the consortium of the EU 7^th^ Framework Project ENPRA. Electron microscopy of these four samples was performed by Scanning Electron Microscopy using a JOEL 7500F Electron Microscope (JOEL Eching, Germany). Representative pictures are shown in Fig. 1. As can be seen in the figure, there were notable contrasts in morphology of the four samples.

For all subsequent experiments, sample ZnO I was selected and referred to as ZnO in this paper. This material has been further characterized in a recent study to address the toxicity of ZnO nanoparticles in rat lungs *in vivo* [53]. Specific characteristics and toxic effects of the other three samples used in this project are described elsewhere [16], [17], [37], [54]. All ZnO samples that were used in the present study were baked at 220°C for 16 h to destroy possible endotoxins.

For the characterization of the agglomeration/aggregation properties of the ZnO particles in the suspensions that were used for cell treatments (described below), dynamic light scattering (DLS) was used. Measurements of the Z.average diameter as well as of the polydispersity index (PDI) of the different ZnO suspensions were performed using a Beckmann Coulter Delsa Nano. The Z.average can be interpreted as harmonic mean of the intensity weighted size distribution and the PDI is a normalised parameter for the variation of the effective hydrodynamic diameter of the sample. As confidence criteria a polydispersity index (PDI) of ≤0.3 was chosen (Z.average comparison is not recommended for values over 0.5). All measurements were conducted directly after preparation of fresh suspensions, or 24 h after the preparation of the suspensions. In addition, DLS was measured for freshly prepared unbaked samples, to evaluate potential agglomeration/aggregation behavior changes due to the heat treatment of the ZnO. Each value was determined by three measurements of two independent preparations (n = 2).

Depending on the type of experiment, cells were seeded in a concentration of 1.1×10^5^/cm^2^ into 6-well plates, 24-well plates, 96-well plates or in 4-chamber-slides (BD Biosciences) and incubated for 24 h prior to treatment. Immediately before each experiment, the ZnO samples were freshly suspended at 0.7 g/L in complete cell culture medium by water bath sonication (60 W, 35 Hz, 10 min, Sonorex TK 52; Bandelin, Berlin, Germany), diluted to the final concentrations of 1, 5, 10, 40 or 80 µg/cm^2^ and treated for 4 h or 24 h as indicated. As positive control, cells were treated with the apoptosis inducer staurosporine (STS, Sigma-Aldrich) at a concentration of 1 µM (4 h) or 0.1 µM (24 h).

### Evaluation of the role of ZnO solubilization

In a specific subset of experiments, the role of solubilization of ZnO nanoparticles in the cell treatment medium was also addressed. Therefore, RAW 264.7 cells were treated either with freshly prepared ZnO suspensions, or with suspensions that were first pre-incubated for 24 h at 37°C. In addition, the effects of the respective particle-free supernatants and pellets of the 24 h pre-incubated suspensions were evaluated, as obtained following 10 min centrifugation at 16.000 *g*.

As an independent approach to evaluate the role of solubilization we employed fluorescence microscopy, with the use of the fluorescent zinc indicator, FluoZinTM-3 (Molecular Probes/Invitrogen). The presence of ionic zinc was evaluated in cells that were treated for 4 h with the aforementioned subset of samples. Cells were seeded in 24-well plates and treated with the respective samples as described above. Every washing step was performed following centrifugation (5 min, 200 g, RT). Cells were rinsed twice with loading buffer (150 mM NaCl, 5 mM KCl, 5 mM Glucose, 1 mM MgCl_2_, 2.2 mM CaCl_2_, 10 mM HEPES, pH 7.4). The staining procedure was performed at a final concentration of 5 mM indicator dye in loading buffer for 30 min at 37°C. Cells were rinsed twice with loading buffer and incubated in the dark for 20 min. After a final washing step, fluorescence was detected using a fluorescence microscope (Zeiss AxioObserver.D1 with AxioCam MRm, controlled by AxioVision Rel. 4.8) at 100× magnification (Carl Zeiss, Oberkochen, Germany).

### Cytotoxicity

Cell viability was determined using the WST-1 conversion assay (Roche, Mannheim, Germany). Care was taken to avoid artefacts in the measurement due to interference of the ZnO particles in this assay, as previously described in detail [33].

### Analysis of DNA fragmentation

Determination of apoptosis was evaluated by chromosomal DNA fragmentation analysis after staining with the DNA-intercalating dye 7-aminoactinomycin D (7-AAD, Sigma-Aldrich). Details of the procedure, which included particle gating, was done as previously described [33]. Apoptotic DNA fragmentation was also detected by using a DNA Fragmentation Imaging Kit (Roche) following the manufacturer's instruction. Based on the TUNEL reaction using terminal deoxynucleotidyl transferase and fluorescein-labeled dUTP, fluorescence detection of cells with apoptotic DNA strand breaks was performed. To examine total cell numbers, nuclei were labeled simultaneously with Hoechst 33342. Merged images of both channels were shown using a fluorescence microscope (Zeiss AxioObserver.D1 with AxioCam MRm, controlled by AxioVision Rel. 4.8) at 100× magnification (Carl Zeiss, Oberkochen, Germany).

### Oxidative DNA damage measurement by the fpg-modified comet assay

The alkaline comet assay was used to measure DNA strand breaks in RAW 264.7 macrophages, with inclusion of the enzyme formamidopyrimidine glycosylase (fpg) to specifically determine oxidative DNA damage. This was done using the method as described by Speit et al. [55] with modifications as specified in Gerloff et al. [54]. Fpg-enzyme was kindly provided by Dr. Andrew Collins, Institute for Nutrition Research, University of Oslo, Norway. As a positive control, oxidative DNA damage was induced by treatment with the photosensitizer Ro 19-8022 ([R]-1-[(10-chloro-4-oxo-3-phenyl-4H-benzo[a]quinolizin-1-yl)carbonyl]-2-pyrrolidinemethanol) (Roche, Basel, Switzerland) after 2 min of light irradiation [35]. DNA strand breakage data are presented as % comet tail values (in the absence of fpg). Oxidative DNA damage is calculated and shown as the differences in % tail DNA as measured in the presence or absence of the Fpg enzyme: delta (Δ)Fpg = [% tail DNA_+Fpg_]−[% tail DNA_-Fpg_].

### Transmission electron microscopy (TEM)

TEM was used to evaluate ultrastructural hallmarks of cell death in RAW 264.7 macrophages. Therefore, cells were treated in 6-well plates, harvested by scraping and washed with PBS (200 g, 10 min). The pellet was fixed with 0.1 M phosphate-buffer (pH 7.4)/2.5% glutaraldehyde/4% paraformaldehyde, stained *en bloc* with uranyl acetate, and embedded in epoxy resin. Ultra-thin sections were cut and examined with a transmission electron microscope (Hitachi H-600, Hitachi High-Technologies Europe GmbH, Krefeld, Germany).

### Immunofluorescence Analysis

Cells were grown and treated in four chamber culture slides (BD Biosciences). All following steps were performed at RT unless otherwise noted and kept separate by three washing steps each for 5 min in PBS. Cells were fixed in 4% paraformaldehyde/PBS (pH 7.4) for 20 min and permeabilised in 0.1% Triton X-100/PBS. Non-specific binding sites were saturated during 1 h incubation in 5% normal goat serum/PBS (Vector Laboratories, Burlingame, CA, USA). Primary antibody against cleaved caspase-3 was diluted 1∶200 in PBS (rabbit monoclonal, Cell Signaling, Boston, MA, USA) and incubated at 4°C overnight in a humid box. The secondary antibody (MFP488 goat anti-rabbit, MoBiTec, Göttingen, Germany) was applied for 1 h 1∶200 in PBS. Nuclear staining was performed by using the dye Hoechst 33342 (Cell Signaling) in a final concentration of 1 µg/ml in PBS for 15 min at 37°C in a humid box. In order to control for unspecific staining primary antibody was replaced by rabbit IgG in the same concentration (Vector Laboratories).

Analysis was performed using a fluorescence microscope (Olympus BX60, Germany) connected with a color camera (U-CMAD3, Olympus) controlled by an image analysis system (analySIS, Olympus, Hamburg, Germany) at 400× magnification.

### Measurement of particle uptake

Cells containing particles may change their granularity, which is directly correlated to the sideward scatter (SSC) measured by FACS analysis. Particle uptake was measured in treated primary macrophages from p47^phox−/−^ and wt mice upon discrimination of free ZnO particles from the cellular events as described previously [33]. After 3 h treatment, cells were washed and suspended in ice-cold HBSS−/− (Invitrogen, Karlsruhe, Germany) and analysed using FACS Calibur (BD Biosciences) Univariant histograms of SSC provided the median of cell granularity used as indicator for particle uptake.

### Superoxide detection via lucigenin-amplified chemiluminescence (LucCL)

For chemiluminescence detection, cells were seeded at 3×10^5^/50 µl in HBSS^+/+^ (Invitrogen) in white 96-well microtiter-plates (BD Biosciences). After particle treatment (50 µl) followed by addition of 100 µl lucigenin (1 g/l in HBSS^+/+^, Fluka, Seelze, Germany) measurement started immediately over 90 min with the luminometer (MicroLumat Plus Microplate Luminometer LB 96 V, EG&G Berthold, Bad Wildbach, Germany).

### Statistical analysis

Data were expressed as mean ± standard deviation of the number of independent experiments as indicated in the figure legends. Statistical analysis was performed employing SPSS 20.0 for windows using ANOVA with Dunnett post-hoc comparison for all assays, except for the comet assay, for which LSD post-hoc testing was employed. Effects on statistical significance were marked by up to three asterisks (*, **, ***) representing significance at cut-off levels of p≤0.05, p≤0.01, and p≤0.001, respectively.
